# Light Stress after Heterotrophic Cultivation Enhances Lutein and Biofuel Production from a Novel Algal Strain *Scenedesmus obliquus* ABC-009

**DOI:** 10.4014/jmb.2108.08021

**Published:** 2021-09-28

**Authors:** Hyun Gi Koh, Yong Tae Jeong, Bongsoo Lee, Yong Keun Chang

**Affiliations:** 1 Department of Chemical and Biomolecular Engineering, Korea Advanced Institute of Science and Technology (KAIST), Daejeon 34141, Republic of Korea; 2 Advanced Biomass R&D Center (ABC), 291 Daehak-ro, Yuseong-gu, Daejeon 34141, Republic of Korea; 3 Department of Animal and Plant Research, Nakdonggang National Institute of Biological Resources (NNIBR), SangJu 37242, Republic of Korea; 4 Department of Microbial Biotechnology, College of Science and Technology, Mokwon University, Daejeon 35349, Republic of Korea

**Keywords:** *Scenedesmus*, lutein, phylogeny, biofuel, raceway pond cultivation

## Abstract

*Scenedesmus obliquus* ABC-009 is a microalgal strain that accumulates large amounts of lutein, particularly when subjected to growth-limiting conditions. Here, the performance of this strain was evaluated for the simultaneous production of lutein and biofuels under three different modes of cultivation – photoautotrophic mode using BG-11 medium with air or 2% CO_2_ and heterotrophic mode using YM medium. While it was found that the highest fatty acid methyl ester (FAME) level and lutein content per biomass (%) were achieved in BG-11 medium with CO_2_ and air, respectively, heterotrophic cultivation resulted in much higher biomass productivity. While the cell concentrations of the cultures grown under BG-11 and CO_2_ were largely similar to those grown in YM medium, the disparity in the biomass yield was largely attributed to the larger cell volume in heterotrophically cultivated cells. Post-cultivation light treatment was found to further enhance the biomass productivity in all three cases and lutein content in heterotrophic conditions. Consequently, the maximum biomass (757.14 ± 20.20 mg/l/d), FAME (92.78 ± 0.08 mg/l/d), and lutein (1.006 ± 0.23 mg/l/d) productivities were obtained under heterotrophic cultivation. Next, large-scale lutein production using microalgae was demonstrated using a 1-ton open raceway pond cultivation system and a low-cost fertilizer (Eco-Sol). The overall biomass yields were similar in both media, while slightly higher lutein content was obtained using the fertilizer owing to the higher nitrogen content.

## Introduction

Microalgae are considered a good source of biomass for the production of renewable energy that can replace fossil fuels over plant-derived biomass. The biofuels produced from microalgae are carbon neutral as microalgae consume the same amount of CO_2_ during cultivation and they can be directly used in existing facilities. Compared to the 1^st^ and 2^nd^ generation biofuels from woods and crops, microalgae-derived biofuels are advantageous in terms of land usage, cultivation periods, and ethical issues [1, 2]. However, algae-derived biofuels are not considered economically feasible, as the current prices of microalgae products remain higher than those of conventional sources [3]. Hence, efforts to reduce production costs have been made in each process, including strain development, cultivation, harvesting, and conversion. In recent years, the utilization of algae-derived materials to produce high-value products such as bio-jet fuel, cosmetics, and nutraceuticals has been studied worldwide to gain economic feasibility.

*Scenedesmus* is one of the most common algal species in freshwater and marine systems worldwide [4]. Several species have been investigated for the production of biofuels [5, 6], and some have been examined as potential candidates for production of lutein, which is one of the main photosynthetic pigments in nature [7-10]. Lutein is well characterized for its important role in maintaining eye health and strong antioxidant properties [11]. Since humans lack essential enzymes to synthesize lutein, sufficient amounts of lutein must be ingested from species such as plants, algae, bacteria, and certain fungi. In particular, as the use of computers and smartphones has increased, the importance of lutein for the protection of the retina has also increased. Currently, marigold petals are the main source of lutein production, which requires a lot of labor, land, water, and time for cultivation and harvesting [12, 13]. To overcome these disadvantages, microalgae are emerging as a potential candidate to replace marigold petals for lutein production. The lutein content in microalgae is 3–6 times higher than that in marigold petals and microalgae can be grown in any region year-round [12]. Various microalgal species such as *Chlamydomonas*, *Chlorella*, *Muriellopsis*, and *Scenedesmus* have been examined for their lutein production rate, and the highest lutein yield of 7.62 mg/l/day was reported in *Chlorella sorokiniana* using two-stage mixotrophic cultivation [14]. However, most studies were restricted to lab-scale cultivation, and there is a lack of actual investigation on large-scale cultivation for industrial applications.

In this study, we isolated a novel strain of *Scenedesmus obliquus* ABC-009, and investigated the potential of lutein production by modulating cultivation mode. Our finding provides important insights for lutein production that meet the increasing demand in industrial field.

## Materials and Methods

### Isolation of *Scenedesmus obliquus* ABC-009

Algal samples were collected from a river in Namwon, Jeon-ra-do, South Korea, and concentrated via centrifugation. The upper part of the centrifuged debris was removed, and the remaining cells were resuspended in distilled water to pick up single cells using a Pasteur’s pipette and microscope. The selected cells were then incubated under light conditions (120 μmol photons/m^2^/s) in TAP (Tris-Acetate-Phosphate) media containing 20 μg/ml ampicillin for 10 days. After incubation, each inoculum was streaked onto TAP agar plates containing ampicillin (100 μg/ml).To ensure the axenic condition of each algal strain, single colonies were serially diluted and streaked onto the same agar plates until single pure algal colonies were obtained. After confirming seven pure algal isolates, we cultivated the strains in TAP medium for strain characterization. Among the seven strains, one of the isolates (ABC-009) turned into an orange-like color at the end of cultivation, while the others remained green. Hence, we analyzed the pigment composition using high-performance liquid chromatography (HPLC) and performed further analysis.

### DNA Sequencing and Phylogenetic Analyses

To obtain the 18s rDNA sequence of the novel ABC-009 strain, genomic DNA was extracted from the cultivated cells using InstaGene Matrix (Bio-Rad, USA) according to the user manual. For polymerase chain reaction (PCR) of 18s rDNA, Phusion polymerase (NEB, England) was used with the following forward and reverse primer pairs: 5′-CCTGGTTGATCCTGCCAG-3′, 5′-TTGATCCTTCTGCAGGTTCA-3′. The amplified 18s rDNA gene was then sequenced using the Sanger method (Solgent_co_, Korea) with the same primer sets. By blasting the results at NCBI, we confirmed the novelty of the 18s rDNA sequence, and the sequence was submitted to GenBank with accession number MG971386. For phylogenetic analysis, the 18s rDNA sequence was aligned against the gene sequences from other algal species, including *Scendesmus*, *Vischeria*, *Chlorella*, *Graesiella*, and *Auxenochlorella*. All phylogenetic analyses were performed using CLC main workbench (version 7.7.2) and the maximum likelihood phylogeny method with a starting tree created using the unweighted pair group method. For each method, 1,000 bootstrap replications were performed.

### Lab-Scale Cultivation of Novel *S. obliquus* ABC-009

The *S. obliquus* ABC-009 strain was cultivated under both phototrophic and heterotrophic conditions. For phototrophic cultivation, cells were inoculated in BG-11 media containing 1.5 g/l NaNO_3_, 0.04 g/l K_2_HPO_4_∙2H_2_O, 0.075 g/l MgSO_4_∙7H_2_O, 0.036 g/l CaCl_2_∙2H_2_O, 0.006 g/l citric acid, 0.02 g/l Na_2_CO_3_, 0.006 g/l ferric ammonium citrate, 0.001 g/l Na-EDTA, and 1 ml of trace metal A5. The trace metal A5 solution consisted of 2.86 g/l H_3_BO_3_, 1.81 g/l MnCl_2_∙4H_2_O, 0.22 g/l ZnSO_4_∙7H_2_O, 0.39 g/l NaMoO_4_∙2H_2_O, 0.079 g/l CuSO_4_∙5H_2_O, and 0.05 g/l CoCl_2_∙6H_2_O under light illumination of 120 μmol photons/m^2^/s, where ambient air or 2% CO_2_ was supplied at the rate of 0.5 vvm. Heterotrophic cultivation was carried out in YM (Yeast Malt) medium (YM broth, BD Difco, USA) under dark conditions without any light sources or air supplements. All cultivation was performed under controlled conditions in artificial incubators at 29°C with agitation at 200 rpm. Each cultivation was performed at least twice.

### Large-Scale Cultivation of *S. obliquus* ABC-009 in Raceway Pond

For large-scale cultivation of *S. obliquus* ABC-009, seed culture was first prepared in a 500-ml Erlenmeyer flask, and then successively transferred to a 5-L barrel, and finally to a 1-ton raceway pond. BG-11 media supplemented with 2% CO_2_ (0.5 vvm) was used for flask and barrel scale cultivation. 20 g/l of commercially available water-soluble fertilizer, Eco-Sol (N-P-K: 25-9-18, Dongbu Farm Hannong, South Korea) and BG11 media supplemented with 0.5% vvm of 2% CO_2_ were used for large-scale cultivation in a raceway pond. Semi-continuous cultivation was carried out in a 1-ton open raceway pond, and two-thirds of the algal culture was harvested from the open pond every two weeks. The harvested culture was replaced with the same volume of nutrition-rich growth media, and this experiment was carried out for a period of 2 months.

### Growth Analysis

During cultivation, cell growth was analyzed by referring to cell number and dry cell weight (DCW). For cell counting, 20 μl of culture was loaded onto Cellometer counting chambers (CHT4-002, Nexcelom, USA), which was then analyzed using an automated cell counter (Cellometer Auto X4, Nexcelom). The DCW was calculated by filtering the cells through GF/C filter paper (Whatman, USA), followed by washing with distilled water. The mass of the filter paper was measured before and after filtration under completely dry conditions.

### Fatty Acid Methyl Ester (FAME) Analysis

Harvested cells of *S. obliquus* ABC-009 were converted into a fine powder after lyophilization in a freeze-dryer (FD5508, IlShinBioiBase, Korea). Total lipids were extracted with 2 ml of a chloroform-methanol mixture (2:1, v/v) from 10 mg of prepared samples and subjected to transesterification by reacting with 300 μl of sulfuric acid and 1 ml of methanol at 100°C for 20 min. For the internal standard, heptadecanoic acid (100 mg) dissolved in 200 ml of chloroform was added. To separate the organic phase from the hydrophilic phase containing proteins and carbohydrates, samples were centrifuged at 4,000 rpm after mixing with 1 ml of distilled water. The recovered FAME in the organic phase was then filtered using a 0.20-μm RC-membrane syringe filter (Sartorius Stedim Biotech, Germany). Using gas chromatography (HP 6890, Agilent, USA), FAME was analyzed with an HP-INNOWax polyethylene glycol column (HP 19091 N-213, Agilent).

### Light-Harvesting Pigment Analysis Using HPLC

Light-harvesting pigments were extracted by bead beating cells in the presence of acetone. For this, 10 ml of cultured cells was harvested in 2-ml twist cap tubes (Bertin Technologies, USA), and was supplemented with 0.1-and 0.5-mm zirconia/silica beads. Then, 1.5 ml of acetone was added before bead beating was performed at 6,000 rpm for 40 s with a bead beater (Percellys 24, Bertin Technologies). To ensure complete extraction of pigments, bead beating was performed 10 times for each sample until the cell debris turned colorless. To avoid pigment degradation by heat, the samples were cooled on ice after each step. After filtering through a 0.20-μm RC-membrane syringe filter (Sartorius Stedim Biotech, Germany), the extracts were analyzed using HPLC as described in a previous study [15].

### Morphological Characterization

Morphological characterization of *S. obliquus* was performed according to cell size, shape, and appearance on agar plates. A microscope (Nikon TS100, Japan) was used for visualization, and a Coulter counter (Multisizer 4, Coulter Counter, USA) was used for cell size measurements.

## Results and Discussion

### Phylogenetic and Phenotypic Analysis of Novel Strain *S. obliquus* ABC-009

A total of seven algal isolates were obtained from nature (Namwon, Jeon-ra-do, South Korea) as explained in the Materials and Methods section, and one of the strains that turned into orange-like color after cultivation for 10 days in TAP media, was temporarily designated as ABC-009 (Fig. S1). As the color of algal cells often provides information on the pigment composition [16], we assumed that the strain possesses some types of carotenoids. HPLC analysis verified that the dominant pigment was lutein. Considering that lutein is a high-valued product with the potential to be used in diverse industrial areas, including healthcare products and cosmetics, we further analyzed the unknown ABC-009 strain.

**Fig. 1:**
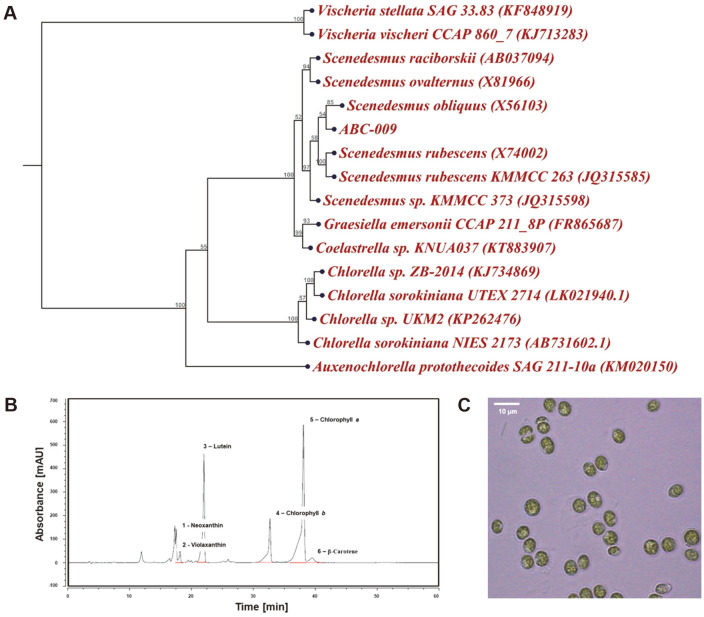
Identification of novel *S. obliquus* ABC-009 strain by A. phylogenetic analysis, B. light-harvesting pigments, and C. morphology. Phylogenetic analysis was based on 18s rDNA gene sequences and bootstrap values are from 1,000 replicates of the sequence data.

The morphology of the ABC-009 strain was first investigated via light microscopy of cells cultivated at 25°C in TAP media. Most mature vegetative cells had spherical or elliptical shapes, with a chloroplast occupying approximately two-thirds of the whole cell (Fig. 1C). The average size of mature vegetative cells was 4.5 × 5 μm. A few vacuoles were observed in the cytoplasm. In general, the shape of *Scenedesmus* sp. is widely known for its unique feature consisting of 4, 8, 16, or 32 cells arranged in a row, with spines or bristles [4]. However, a few *Scenedesmus* species, such as *S. obliquus* or *S. rubescens*, also exhibit spherical shapes similar to the ABC-009 strain, and some are known to change their form upon cultivation conditions [17]. Accordingly, we could not determine the exact algal species of the ABC-009 strain because these features are often found in various species, including *Chlorella*, *Scenedesmus*, and *Ettlia* [18, 19].

The phylogenetic analysis based on the sequence of 18S rDNA, however, could provide more clues on the genus to which it belongs (Fig. 1A). The phylogenetic tree showed clear similarity of the 18S rDNA with most *Scenedesmus* species, especially with *Scenedesmus obliquus*. In addition, as the novel strain contained lutein and neoxanthin as the main xanthophyll pigments, similar to *Scenedesmus* sp. (Fig. 1B), we concluded that the isolated strain was *S. obliquus* ABC-009 after considering overall aspects of the morphology, pigments, and 18s rDNA sequence.

### Cultivation under Different Conditions

Although most microalgae are considered photoautotrophs, heterotrophic cultivation is also adopted for the production of high-value products in several species, including *Scenedesmus* sp. [14, 20]. Often, heterotrophic cultivation has the advantage of better cell growth compared to phototrophic cultivation, allowing higher final cell densities [20]. In contrast, heterotrophic cultivation often results in lower contents of light-harvesting pigments and lipids, which is relevant to the lack of sufficient light. To determine the optimal conditions for producing lutein and lipids from *S. obliquus* ABC-009, we cultivated the microalgae under three different conditions: photoautotrophic cultivation with ambient air, photoautotrophic cultivation with 2% CO_2_, and heterotrophic cultivation with 10 g/l glucose. Through a preliminary study in 96-well plates, the optimal temperature for cultivation was found to be 29°C, where YM media showed the best performance over BG11 or TSB (Tryptic Soy Broth) media (Fig. S2).

**Fig. 2:**
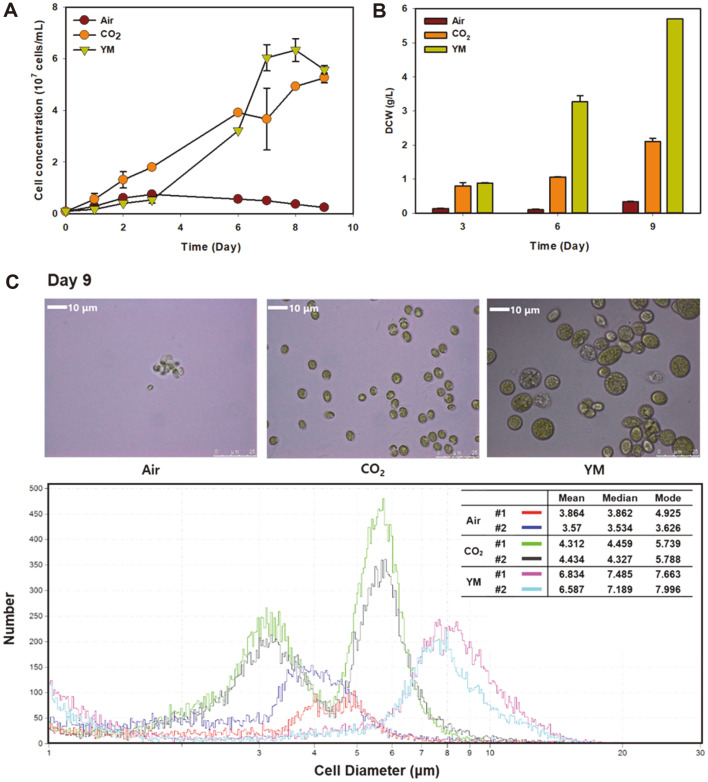
Cultivation of *S. obliquus* ABC-009 in photoautotrophic (air or CO_2_) and heterotrophic conditions. BG-11 and YM media supplemented with 10 g/l glucose were used for each cultivation condition. **A**. The cell density of CO_2_ supplied cells and heterotrophically cultivated cells reached up to nearly 6 × 10^7^ cells/ml, while cells did not grow well with ambient air. **B**. Heterotrophically cultivated cells had much higher DCW, implying changes in composition or morphology. **C**. Heterotrophically grown cells exhibited larger cell sizes compared to those of the photoautotrophically grown cells.

The growth rate according to cell numbers showed similar results in CO_2_ supplemented photoautotrophic cultivation and heterotrophic cultivation, while cultivation with ambient air was insufficient to provide sufficient CO_2_ (Fig. 2A). However, the dry cell weight of heterotrophically cultivated cells was3-fold higher than that of autotrophically cultivated cells (Fig. 2B). As an increase in dry cell weight without any changes in cell density may imply variations in cell morphology or contents, we first analyzed the cells with a microscope and observed significantly increased cell size in heterotrophically cultivated cells (Fig. 2C). The average size of cells was the largest in the order of heterotrophically cultivated cells (6.71 μm), CO_2_ supplemented cells (4.37 μm) and cells cultivated with ambient air (3.72 μm). A similar phenomenon of increased cell size under heterotrophic cultivation was reported in *Chlorella* sp. without clarification of the exact underlying mechanism [21]. Since large cells require less energy for harvesting [22], the cultivation of *S. obliquus* ABC-009 under heterotrophic conditions may be a good option for industrial-scale cultivation. Furthermore, as microalgae with increased cell sizes are reported to exhibit higher transformation efficiency [23], this effect of cell enlargement can be applied for genetic engineering of *S. obliquus* ABC-009 in the future.

### FAME and Lutein Content

**Fig. 3:**
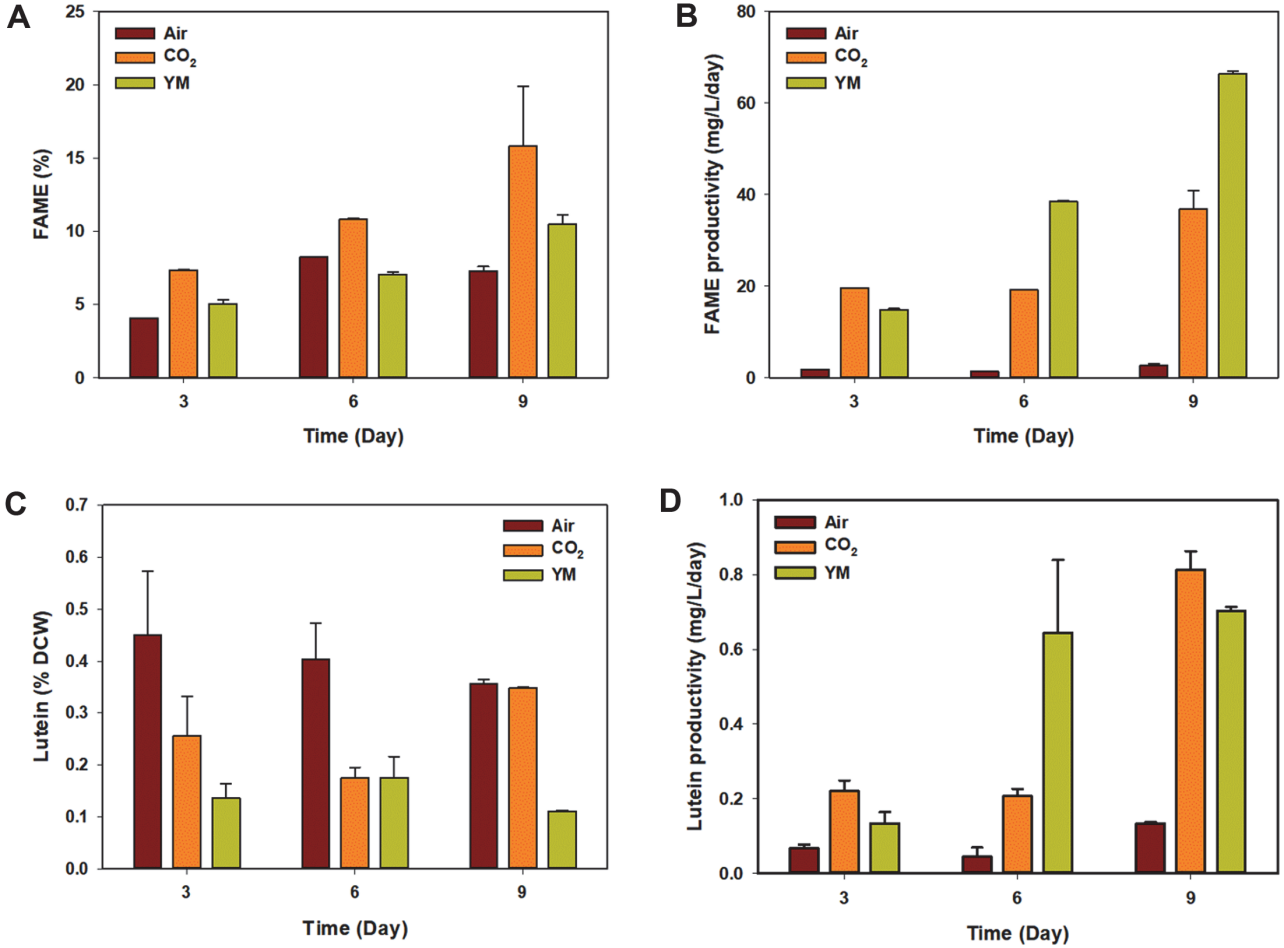
The contents and productivity of FAME and lutein in each condition. **A, C**. FAME and lutein contents were measured from photoautotrophically cultivated cells and compared to those of the cells grown in heterotrophic condition. **B**. Due to the high biomass of heterotrophically cultivated cells, FAME productivity of these cells was calculated on day 9. **D**. Lutein productivity was highest in photoautotrophically cultivated cells with CO_2_ supplementation (day 9). Each data instance represents the mean ± SD of triplicate measurements.

In order to compare the lutein and lipid productivity of cells cultivated under different conditions, pigments and FAME were analyzed using HPLC and GC, respectively (Fig. 3). The highest FAME content was observed in cells cultivated photoautotrophically with CO_2_ supplementation (Fig. 3A). Due to the existence of an organic carbon source, the lipid accumulation was rather limited under heterotrophic conditions, showing even lower FAME contents than the photoautotrophically cultivated cells with ambient air on day 6 (8.2% and 7.1%, respectively). The maximum FAME content in CO_2_ supplemented cells (15.8%) and heterotrophically grown cells (10.5%) was achieved on day 9. However, due to high biomass, the FAME productivity was 1.8 folds higher in heterotrophically cultivated cells (66.38 mg/l/day) than in photoautotrophically cultivated cells with CO_2_ supplementation (36.85 mg/l/day) (Fig. 3B).

The lutein content of cultivated cells was between 0.1% and 0.45% of its biomass, and the highest content was observed on day 3 in the aerobically cultivated cells (0.45%) (Fig. 3C). However, the actual quantity of lutein in aerobically cultivated cells on day 3 was merely 0.03 μg/cell, which was much lower than the cases of cells cultivated with CO_2_ (0.08 μg/cell) or YM media (0.11 μg/cell) (Fig. 3D). Accordingly, the high lutein content in aerobically cultivated cells was only due to the low cell biomass, and as a consequence, the lutein content actually decreased over time under aerobic conditions. In contrast, lutein content increased from 0.25% DCW to 0.35%DCW in CO_2_ supplemented cells, whereas no significant changes were detected in heterotrophically cultivated cells. As photosynthesis does not take place under dark conditions, it was difficult for the lutein, a kind of photosynthetic pigment, to increase above 0.2% of DCW in heterotrophic condition.

### Post-Cultivation Light Stress for FAME and Lutein Accumulation

**Fig. 4:**
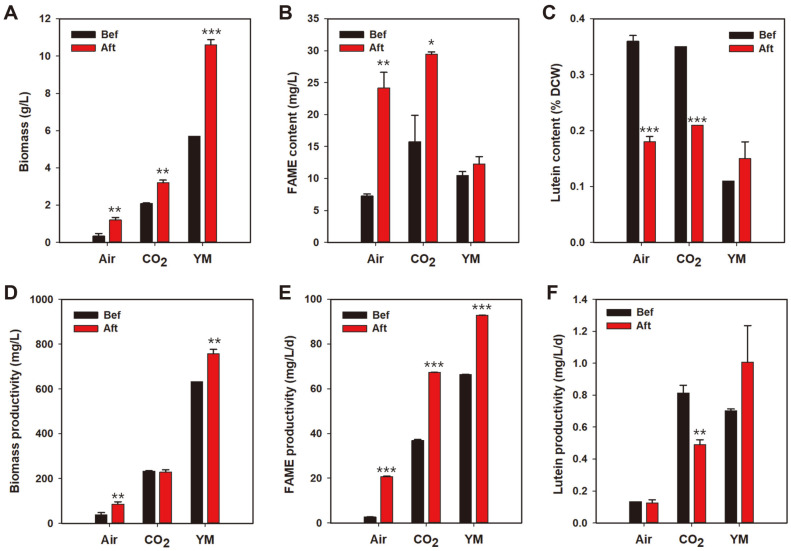
The contents and productivity of biomass, FAME, and lutein, before and after post-cultivation light treatment. The day 9 cells cultivated in each condition were treated with 150 μmol photons/m^2^/s of light without shaking for 5 days. Asterisks indicate the significant differences between before and after treated samples, determined by Student’s t test. (*p<0.05, **p<0.01, ***p<0.001).

Compared to the high biomass productivity under heterotrophic conditions, the productivity of FAME and lutein was not as high as expected because of their low contents. To increase the lutein and FAME contents, fully cultivated cells were exposed to 150 μmol photons/m^2^/s of LED light for 5 days without shaking (Fig. S3). Induction of post-cultivation stress by light, salinity, or nutrient starvation has been studied in a few microalgae to increase the amounts of metabolites [24, 25]. As our target products were lipid and lutein, we decided to increase only light intensity to minimize the extra energy input for post-cultivation stressing. Five days of light irradiation on cells cultivated in each condition resulted in a color change from green to yellow, which may be the result of increased carotenoid content and degradation of chlorophylls. After post-cultivation light stress period, the overall biomass was increased in all three conditions, and the highest increase observed was 4.8 g/l in the heterotrophically cultivated cells (Fig. 4). However, the FAME content of heterotrophically cultivated cells increased by only 17%, while increases of 336% and 86% were measured in photoautotrophically cultivated cells with air or CO_2_, respectively. These results suggest that nutrients were not completely depleted in YM media, and cells continued to grow in a semi-mixotrophic condition, resulting in a large increase in biomass and small changes in lipid content. On the other hand, photoautotrophically cultivated cells face nutritional starvation, causing them to accumulate lipids instead of biomass. Likewise, lutein content was decreased by 39–51% under photoautotrophic conditions, while a 34% increase was observed in heterotrophically grown cells. As heterotrophic cultivation was carried out in a completely dark condition, exposure to light triggered photosynthetic metabolism, including biosynthesis of light-harvesting pigments. In conclusion, we have successfully increased the FAME and lutein productivity by post-cultivation light irradiation, achieving the highest FAME and lutein productivity of 92.78 mg/l/day and 1.006 mg/l/day from heterotrophically cultivated cells. Among the diverse *Scenedesmus* sp. studied for lutein production, the productivity of *S. obliquus* ABC-009 was around the average value (Table 1). However, it is difficult to directly compare the lutein productivity of each strain from different studies as growth conditions (scale, media composition, temperature, light source, extraction method, osmotic stress, etc.) are not the same. Previous studies have revealed many favorable conditions for lutein production in selected *Scenedesmus* species. For example, white LEDs resulted in better production efficiency than other LEDs, with the best performance at 300 μmol photons/m^2^/s [7]. In addition, Chen *et al*. found that mixotrophic condition with a 12 h light period followed by a 12 h dark period can increase lutein productivity [8], and Sanchez *et al*. reported that pH 8 and low salt stress conditions work well for *Scenedesmus almeriensis* [10]. Many variables that may affect lutein productivity remain, and the optimal conditions can vary depending on the specific strain. Hence, further studies on *S. obliquus* ABC-009 are necessary to determine the optimal conditions for maximizing lutein productivity.

### Large-Scale Cultivation of *S. obliquus* ABC-009

**Fig. 5:**
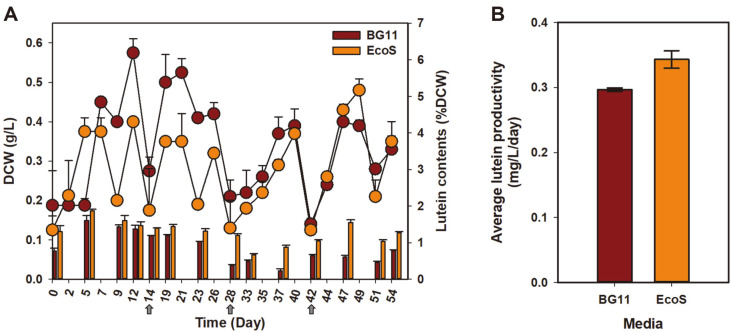
Outdoor raceway pond cultivation of *S. obliquus* ABC-009 using BG-11 and low-cost farm fertilizer Eco-Sol for two months. **A**. DCW (line graph) and lutein contents (bar graph). **B**. Lutein productivity (mg/l/day). Each data instance represents the mean ± SD of duplicates from an independent raceway pond.

In particular, *S. obliquus* is a robust microalga that has stable biomass productivity in moderate climates [28, 29]. Moreover, the stability of scale-up outdoor cultivation of microalgae over long periods is important for the successful commercialization of microalgae-based products [30]. Several previous studies have reported the enhancement of lutein productivity in *Scenedesmus* sp. These include the modulation of cultivation modes, such as two-stage cultivation, mixotrophic and heterotrophic cultivation, and light induction strategies [7-9, 31]. However, studies on large-scale photoautotrophic cultivation using low-cost media for lutein production have rarely been reported. To evaluate the potential of large-scale outdoor cultivation using low-cost media, 1-ton scale outdoor cultivation was carried out for 2 months, and biomass and lutein content, and productivity were analyzed. The results showed that the lutein content of cells grown in Eco-Sol, the low-cost media, was generally much higher (111 ~ 382%) than that in cells grown in BG-11, while biomass production was generally higher in culture using BG-11 (50 ~ 216%) (Fig. 5A). From the comparison of elemental compositions in each medium (Table 2), we can see that Eco-Sol media contained a much higher amount of nitrogen (20x), phosphorous (252x), and potassium (196x) compared to BG-11, while the abundance of other trace elements was similar or richer in BG-11. It has been reported that a sufficient amount of nitrogen is essential for lutein accumulation, and lutein content may depend on residual nitrogen concentration in green algae [32, 33]. Hence, the high lutein content in cells cultivated with Eco-Sol may be due to the higher nitrogen concentration, which was approximately 20 folds of that in BG-11. As a result, overall lutein productivity considering biomass and lutein content over the long-term cultivation was approximately 16% higher in the culture grown using Eco-Sol than that in culture grown using BG-11, indicating that low-cost media employed for stable biomass and lutein production through the large-scale outdoor cultivation of *S. obliquus* ABC-009 (Fig. 5B).

## Supplemental Materials

Supplementary data for this paper are available on-line only at http://jmb.or.kr.

## Figures and Tables

**Table 1:** Biomass and lutein productivity of several *Scenedesmus* sp. from published papers.

Species	Culture condition	Biomass productivity (mg/l/d)	Lutein content (mg/g)	Lutein productivity (mg/l/d)	Ref.
*Scenedesmus obliquus* ABC-009	Photoautotrophic	233.33	3.5	0.813	This study
*Scenedesmus obliquus* ABC-009	Heterotrophic	757.14	1.47	1.006	This study
*Scenedesmus bijugus* (Ladakh)	Photoautotrophic	174.77	2.9	0.47	Minhas *et al*. [26]
*Scenedesmus* sp. (P152)	Photoautotrophic	144.08	1.8	0.24	Minhas *et al*. [26]
*Scenedesmus incrassatulus*	Two-stage heterotrophic	2610	1.49	3.10	Saha *et al*. [9]
*Scenedesmus* sp. CCNM 1028	Photoautotrophic (Optimized media)	472.25	2.12	1.001	Ram *et al*. [27]
*Scendesmus obliquus* CWL-1	Mixotrophic	820	1.78	1.43	Chen *et al*. [8]
*Scendesmus almeriensis*	Photoautotrophic	630	4.3	2.709	Sanchez *et al*. [10]

**Table 2:** Comparison of elemental compositions in BG-11 and Eco-Sol media. (Unit: g/l)

	N	P	K	Mg	B	Fe	Mn	Zn	Cu	Mo	S	Co	Ca
BG-11	0.2503	0.0071	0.0184	0.0122	0.4996	0.0007	0.5030	0.0505	0.0201	0.1547	0.0446	0.0100	0.0098
Eco-Sol	5.0000	1.8000	3.6000	0.1200	0.0100	0.0050	0.0050	0.0016	0.0015	0.0001	n.d	n.d	n.d

## References

[1] Lam MK, Lee KT (2012). Microalgae biofuels: a critical review of issues, problems and the way forward. Biotechnol. Adv..

[2] Torres CM, Rios SD, Torras C, Salvado J, Mateo-Sanz JM, Jimenez L (2013). Microalgae-based biodiesel: a multicriteria analysis of the production process using realistic scenarios. Bioresour. Technol..

[3] Fasaei F, Bitter JH, Slegers PM, van Boxtel AJB (2018). Techno-economic evaluation of microalgae harvesting and dewatering systems. Algal Res..

[4] Lürling M (1999). The smell of water: grazer-induced colony formation in *Scenedesmus*.

[5] Mandal S, Mallick N (2009). Microalga *Scenedesmus obliquus* as a potential source for biodiesel production. Appl. Microbiol. Biotechnol..

[6] Ho SH, Chen WM, Chang JS (2010). *Scenedesmus obliquus* CNW-N as a potential candidate for CO_2_ mitigation and biodiesel production. Bioresour. Technol..

[7] Ho SH, Chan MC, Liu CC, Chen CY, Lee WL, Lee DJ (2014). Enhancing lutein productivity of an indigenous microalga *Scenedesmus obliquus* FSP-3 using light-related strategies. Bioresour. Technol..

[8] Chen W-C, Hsu Y-C, Chang J-S, Ho S-H, Wang L-F, Wei Y-H (2019). Enhancing production of lutein by a mixotrophic cultivation system using microalga *Scenedesmus obliquus* CWL-1. Bioresour. Technol..

[9] Florez-Miranda L, Canizares-Villanueva RO, Melchy-Antonio O, Martinez-Jeronimo F, Flores-Ortiz CM (2017). Two stage heterotrophy/photoinduction culture of *Scenedesmus incrassatulus*: potential for lutein production. J. Biotechnol..

[10] Sánchez JF, Fernández JM, Acién FG, Rueda A, Pérez-Parra J, Molina E (2008). Influence of culture conditions on the productivity and lutein content of the new strain *Scenedesmus almeriensis*. Process Biochem..

[11] Kijlstra A, Tian Y, Kelly ER, Berendschot TTJM (2012). Lutein: more than just a filter for blue light. Prog. Retin. Eye Res..

[12] Lin JH, Lee DJ, Chang JS (2015). Lutein production from biomass: marigold flowers versus microalgae. Bioresour. Technol..

[13] Saha SK, Ermis H, Murray P (2020). Marine microalgae for potential lutein production. Appl. Sci..

[14] Chen CY, Liu CC (2018). Optimization of lutein production with a two-stage mixotrophic cultivation system with *Chlorella sorokiniana* MB-1. Bioresour. Technol..

[15] Koh HG, Kang NK, Jeon S, Shin SE, Jeong BR, Chang YK (2019). Heterologous synthesis of chlorophyll b in *Nannochloropsis salina* enhances growth and lipid production by increasing photosynthetic efficiency. Biotechnol. Biofuels.

[16] Tuli HS, Chaudhary P, Beniwal V, Sharma AK (2015). Microbial pigments as natural color sources: current trends and future perspectives. J. Food Sci. Technol..

[17] Mercado I, Alvarez X, Verduga ME, Cruz A (2020). Enhancement of biomass and lipid productivities of *Scenedesmus* sp. cultivated in the wastewater of the dairy industry. Processes.

[18] Safi C, Zebib B, Merah O, Pontalier PY, Vaca-Garcia C (2014). Morphology, composition, production, processing and applications of *Chlorella vulgaris*: a review. Renew. Sust. Energ. Rev..

[19] Pegg C, Wolf M, Alanagreh La, Portman R, Buchheim MA (2015). Morphological diversity masks phylogenetic similarity of *Ettlia* and *Haematococcus* (Chlorophyceae). Phycologia.

[20] Kamalanathan M, Chaisutyakorn P, Gleadow R, Beardall J (2018). A comparison of photoautotrophic, heterotrophic, and mixotrophic growth for biomass production by the green alga *Scenedesmus* sp. (Chlorophyceae). Phycologia.

[21] Kim DG, Hur SB (2013). Growth and fatty acid composition of three heterotrophic *Chlorella* species. Algae.

[22] Aligata AJ, Tryner J, Quinn JC, Marchese AJ (2019). Effect of microalgae cell composition and size on responsiveness to ultrasonic harvesting. J. Appl. Phycol..

[23] Jeon S, Kang NK, Suh WI, Koh HG, Lee B, Chang YK (2019). Optimization of electroporation-based multiple pulses and further improvement of transformation efficiency using bacterial conditioned medium for *Nannochloropsis salina*. J. Appl. Phycol..

[24] Poh ZL, Amalina Kadir WN, Lam MK, Uemura Y, Suparmaniam U, Lim JW (2020). The effect of stress environment towards lipid accumulation in microalgae after harvesting. Renew. Energy.

[25] Shokravi Z, Shokravi H, Chyuan OH, Lau WJ, Koloor SSR, Petrů M (2020). Improving 'lipid productivity' in microalgae by bilateral enhancement of biomass and lipid contents: a review. Sustainability.

[26] Minhas AK, Hodgson P, Barrow CJ, Sashidhar B, Adholeya A (2016). The isolation and identification of new microalgal strains producing oil and carotenoid simultaneously with biofuel potential. Bioresour. Technol..

[27] Ram S, Paliwal C, Mishra S (2019). Growth medium and nitrogen stress sparked biochemical and carotenogenic alterations in *Scenedesmus* sp. CCNM 1028. Bioresour. Technol. Rep..

[28] Batista AP, Moura P, Marques P, Ortigueira J, Alves L, Gouveia L (2014). *Scenedesmus obliquus* as feedstock for biohydrogen production by *Enterobacter aerogenes* and *Clostridium butyricum*. Fuel.

[29] Gouveia L, Oliveira AC (2009). Microalgae as a raw material for biofuels production. J. Ind. Microbiol. Biotechnol..

[30] Feng P, Yang K, Xu Z, Wang Z, Fan L, Qin L (2014). Growth and lipid accumulation characteristics of *Scenedesmus obliquus* in semi-continuous cultivation outdoors for biodiesel feedstock production. Bioresour. Technol..

[31] Chan MC, Ho SH, Lee DJ, Chen CY, Huang CC, Chang JS (2013). Characterization, extraction and purification of lutein produced by an indigenous microalga *Scenedesmus obliquus* CNW-N. Biochem. Eng. J..

[32] Xie Y, Zhao X, Chen J, Yang X, Ho SH, Wang B (2017). Enhancing cell growth and lutein productivity of *Desmodesmus* sp. F51 by optimal utilization of inorganic carbon sources and ammoniumsalt. Bioresour. Technol..

[33] Chen CY, Ho SH, Liu CC, Chang JS (2017). Enhancing lutein production with *Chlorella sorokiniana* Mb-1 byoptimizing acetate and nitrate concentrations under mixotrophic growth. J. Taiwan Inst. Chem. Eng..

