# Supplementary material for: Light Stress after Heterotrophic Cultivation Enhances Lutein and Biofuel Production from a Novel Algal Strain Scenedesmus obliquus ABC-009
Source: J Microbiol Biotechnol. 2021 Sep 28;32(3):378–86. doi: 10.4014/jmb.2108.08021 (PMC9628787; doi:10.4014/jmb.2108.08021)

J. Microbiol. Biotechnol.
https://doi.org/10.4014/jmb.2108.08021

Supplementary Materials

Post-cultivational light stress enhances lutein and biofuel production from *Scenedesmus obliquus* ABC-009 available for large-scale cultivation with low-cost farm fertilizer

Figure S1. Isolation and identification of *Scenedesmus obliquus* ABC-009 by turning color among seven microalgae isolates.

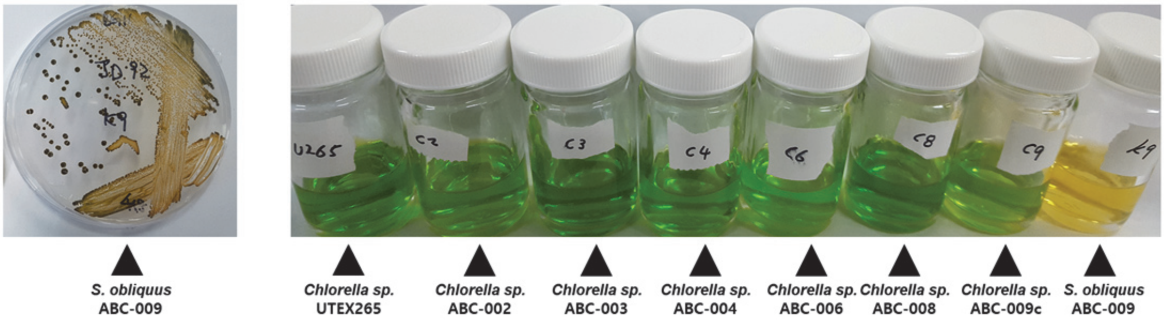

Figure S2. Optimal culture conditions of *Scenedesmus obliquus* ABC-009.

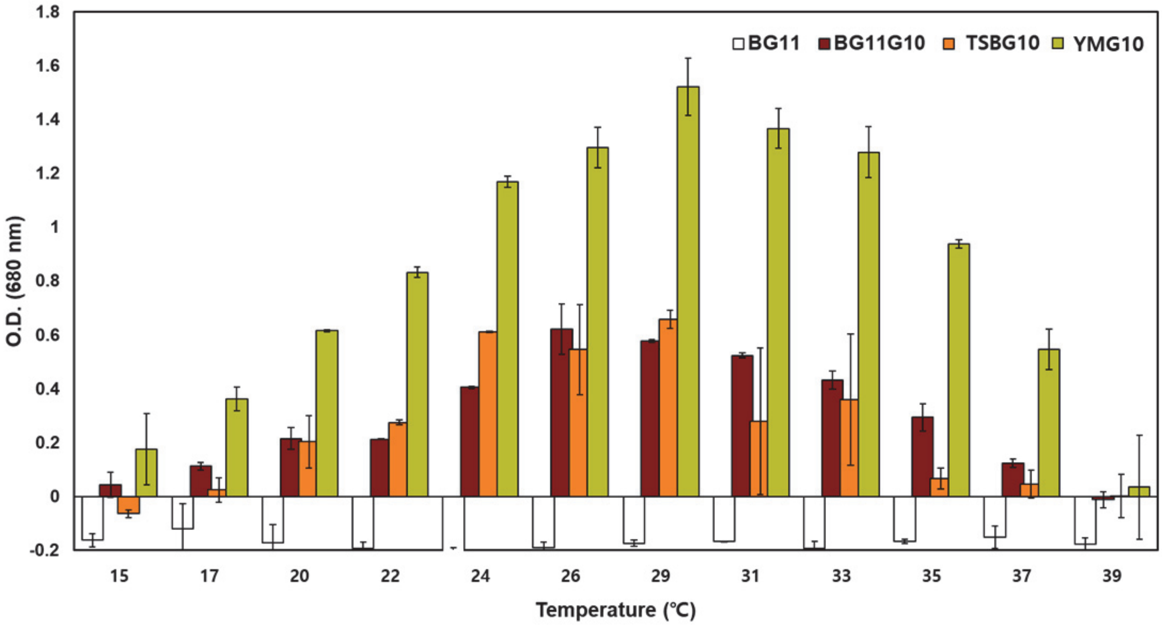

Figure S3. Light induction strategies for enhancing lutein productivity.

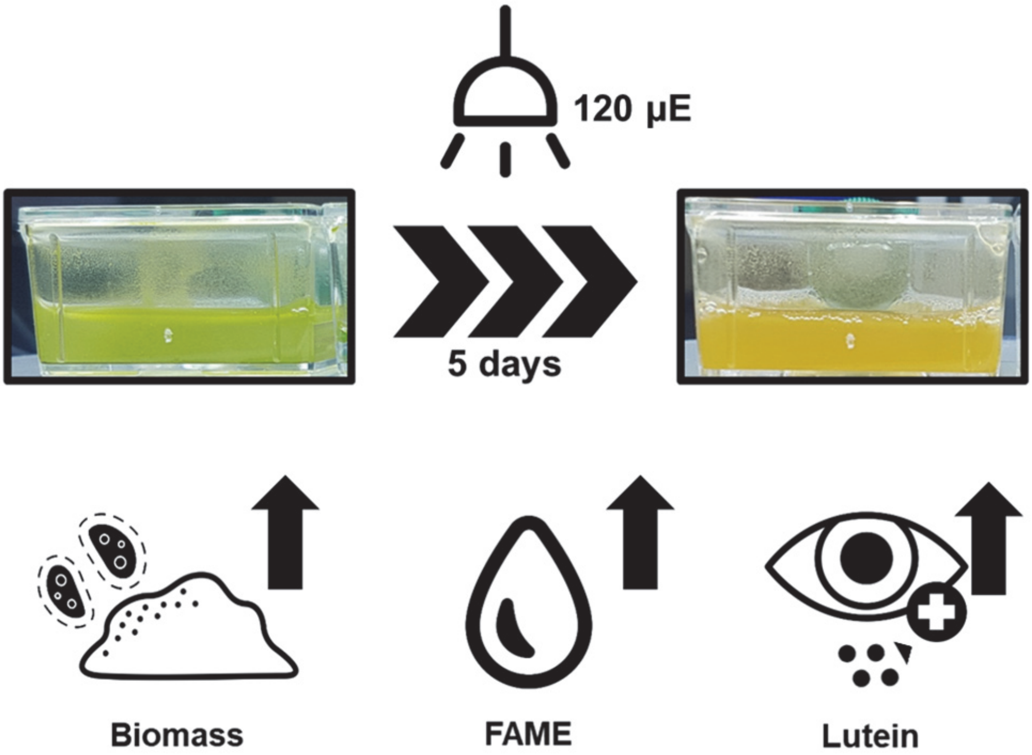

Supplement: Supplementary file 1 [file jmb-32-3-378-supple.pdf]
